# Evaluation of Inertial Sensor-Based Pre-Impact Fall Detection Algorithms Using Public Dataset

**DOI:** 10.3390/s19040774

**Published:** 2019-02-13

**Authors:** Soonjae Ahn, Jongman Kim, Bummo Koo, Youngho Kim

**Affiliations:** Department of Biomedical Engineering, Yonsei University, Wonju 26493, Korea; asj8652@yonsei.ac.kr (S.A.); jmkim0127@ybrl.yonsei.ac.kr (J.K.); bmk726@ybrl.yonsei.ac.kr (B.K.)

**Keywords:** ADLs, fall detection algorithm, falls, IMU, lead time, public dataset

## Abstract

In this study, pre-impact fall detection algorithms were developed based on data gathered by a custom-made inertial measurement unit (IMU). Four types of simulated falls were performed by 40 healthy subjects (age: 23.4 ± 4.4 years). The IMU recorded acceleration and angular velocity during all activities. Acceleration, angular velocity, and trunk inclination thresholds were set to 0.9 g, 47.3°/s, and 24.7°, respectively, for a pre-impact fall detection algorithm using vertical angles (VA algorithm); and 0.9 g, 47.3°/s, and 0.19, respectively, for an algorithm using the triangle feature (TF algorithm). The algorithms were validated by the results of a blind test using four types of simulated falls and six types of activities of daily living (ADL). VA and TF algorithms resulted in lead times of 401 ± 46.9 ms and 427 ± 45.9 ms, respectively. Both algorithms were able to detect falls with 100% accuracy. The performance of the algorithms was evaluated using a public dataset. Both algorithms detected every fall in the SisFall dataset with 100% sensitivity). The VA algorithm had a specificity of 78.3%, and TF algorithm had a specificity of 83.9%. The algorithms had higher specificity when interpreting data from elderly subjects. This study showed that algorithms using angles could more accurately detect falls. Public datasets are needed to improve the accuracy of the algorithms.

## 1. Introduction

Falls are a significant cause of injury and death among the elderly [1] and are defined as events that result in a person coming to rest inadvertently on the ground or another lower level [2]. The risk of falls increases for the elderly due to lower physical strength [3]. Elderly people have stiffer, less coordinated, and more dangerous gaits than the young. Posture control, body-orienting reflexes, muscle strength and tone, and the height of stepping all deteriorate with aging and impair the ability to avoid a fall after an unexpected trip or slip [3]. As old age progresses, the strategy for maintaining balance after a slip shifts from a rapid-correcting “hip strategy” (fall avoidance via weight shifts at the hip) to a “step strategy” (fall avoidance via a rapid step), and then, to a total loss of ability to make a correction in time to prevent a fall [3]. Age-associated impairments of vision, hearing, and memory also tend to increase the number of trips and stumbles [3]. According to the World Health Organization (WHO) [4], the frequency of falls increases with an increase in age and frailty, and the risk of fractures increases exponentially with age due to decreased bone density.

Fall-prevention strategies involve identifying individuals with an increased risk of falling and implementing the appropriate prevention mechanisms. Fall-risk assessments are undertaken using functional tests and questionnaires. Functional tests, such as Time Up and Go (TUG) [5], the Berg Balance Scale [6], and the Performance Oriented Mobility Assessment [7] are objective and used in clinical settings for prospective fall-risk analysis. Based on the results of these evaluation methods, some researchers have tried to prevent falls through exercise prescription or new training using yoga, Pilates, and other balancing exercises [8,9,10]. In real life, most falls are caused by a sudden loss of balance due to an unexpected slip or trip, or by a loss of stability during movements such as turning, bending, and rising [11]. Therefore, those methods cannot completely prevent falls. 

Because falls cannot be completely prevented, protection devices are required to mitigate the impact and avoid hip fractures when falls occur. However, elderly persons are not likely to wear them because they hinder their behavior [12]. Such pads have been reported to move within the clothes and cannot adequately protect the impact area when a fall occurs [13]. To overcome these problems and prevent or reduce injuries due to falls, it is necessary to detect falls during the fall’s descent (pre-impact fall detection) and mitigate the impact using other forms of the protection system. Several groups have developed methods to detect falls before impact [14,15,16,17,18,19,20,21]. Wu implemented a pre-impact fall detection algorithm using thresholds for horizontal and vertical trunk velocities based on three-dimensional (3D) motion capture measurements [16]. She showed that falls could be distinguished from activities of daily living (ADL) with a 300–400 ms lead time before impact.

A wearable sensor was used in many studies to measure inertial properties during falls [15,17,18,19]. Bourke et al. [15] developed a pre-impact fall detection algorithm based on a threshold of vertical trunk velocity. A motion capture system and an inertial sensor on the chest, consisting of a tri-axial accelerometer and a triaxial gyroscope, were used. Falls were distinguished from ADLs with 100% accuracy and were detected an average of 323 ms before trunk impact and 140 ms before knee impact. Other studies have used algorithms, such as support vector machine (SVM) [20] and Markov models [21], to detect falls. Thus far, these systems cannot respond to falls in real-time. If a fall can be detected in its earliest stage during descent, a more efficient impact reduction can be implemented using the longer lead time [1].

Many fall detection systems have been developed [14,15,16,17,18,19,20,21], but developers have faced several challenges. First, most systems were not tested with elderly subjects, reducing their accuracy in real-life applications [21]. Second, all publicly available datasets about falls exclusively contain data from younger subjects, making them less valuable for testing new algorithms [22]. These datasets only contain data from younger subjects because it is risky to try to collect fall data from elderly subjects.

New strategies for overcoming these challenges require testing and acquiring datasets with data collected from elderly subjects about common types of falls and ADLs. To this end, some researchers have analyzed how elderly subjects fall [23,24]. Most activities used to test fall detection algorithms are based on these elderly fall case studies [23,24]. Many researchers have made datasets publicly available for testing fall detection algorithms [25,26,27,28,29], including SisFall [29], MobiFall [25], tFall [26], DLR [27], and Project gravity [28]. The SisFall dataset includes data from 23 younger subjects between 19 and 30 years old and 15 elderly subjects performing 19 ADLs, and 15 falls selected based on the results of a survey and examining those used in previous studies. It includes acceleration and angular velocity data. The dataset and videos of each activity performed by the subjects are available for download for free [29].

In this study, pre-impact fall detection algorithms were developed using inertial measurement unit (IMU) sensors placed on the subjects’ waists. The algorithms used subjects’ vertical angles (Vas) and triangle features (TFs), which are features of tilting. The subjects engaged in four types of simulated falls and six types of ADLs to validate the pre-impact fall detection algorithms developed in this study. The performance of the algorithm using VAs and TF was evaluated using the SisFall dataset [29].

## 2. Materials and Methods

### 2.1. Subjects

A total of 40 healthy male subjects (age: 23.4 ± 4.4 years, weight: 68.7 ± 8.9 kg, height: 172.0 ± 7.1 cm) participated in this study. None of the subjects had any neuromusculoskeletal conditions. The experimental protocol was approved by the Yonsei University Research Ethics Committee (1041849-201308-BM-001-01), and prior written informed consent for participating in the study was obtained from each subject.

### 2.2. Equipment

**Figure 1:**
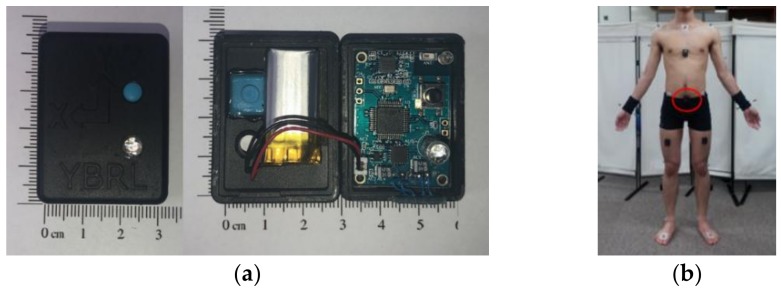
(**a**) Inertial Measurement Unit (IMU) sensor; (**b**) Sensor position.

An MPU-9150 (InvenSense, San Diego, CA, USA) motion-tracking device containing a 3-axis accelerometer and a 3-axis gyro-sensor was used to develop a pre-impact fall detection algorithm [30]. The IMU sensor (Figure 1a) measured acceleration and angular velocity and wirelessly sent data to the data receiver. The data receiver was connected to a computer to store and analyze the data. The sensor was attached to the middle of the left and right anterior superior iliac spines (Figure 1b) in order to provide high distinction among activities with a single accelerometer system [25]. IMU sensor data was sampled at 100 Hz. All falls and ADLs were recorded using a Bonita motion capture camera (Vicon Motion Systems Ltd., Oxford, UK) at 340 frames/s.

### 2.3. Experimental Procedures

All subjects performed ADL movements, which included sitting-to-standing, standing-to-sitting, and sitting-to-lying transitions, walking, jumping, and running (Table 1). A stool and a mattress were used during the ADL trials. The younger subjects performed all ADLs, but the elderly subjects did not conduct the jumping or running trials due to their increased risk of injury. Each subject performed each assigned activity three times. For the falling simulations, subjects were asked to stand on the floor beside a soft foam mattress, and then to fall as if they were fainting forward, backward, backward while twisting by rotating horizontally (twist fall), and laterally (Table 1). Only the younger subjects performed the fall simulations five times.

### 2.4. Data Analysis

Data analysis was performed using MATLAB R2010a (MathWorks Inc., Natick, MA, USA). The sum of the acceleration vectors, pitch, and roll angular velocity was used to develop the pre-impact fall detection algorithms. The total sum vector of the acceleration, $ACC_{\mathrm{SVM}}$, was calculated from the resampled data, as shown in Equation (1):

(1) $$ACC_{\mathrm{SVM}}=\sqrt{ACC_{x}{}^{2}+ACC_{y}{}^{2}+ACC_{z}{}^{2}}$$

where $ACC_{x}$, $ACC_{y}$, and $ACC_{z}$ are the acceleration in the x-, y-, and z-axes, respectively.

The total sum vector, $\omega_{\mathrm{SVM}}$, was calculated from the resampled data, as shown in Equation (2):

(2) $$\omega_{\mathrm{SVM}}=\sqrt{\omega_{\mathrm{Pitch}}{}^{2}+\omega_{\mathrm{Roll}}{}^{2}}$$

where $\omega_{\mathrm{Pitch}}$ and $\omega_{\mathrm{Roll}}$ are the angular velocities in the pitch and roll, respectively.

Acceleration data were transformed into trunk inclinations in the sagittal and frontal planes, measuring how many degrees these body segments deviated from the vertical axis, such that standing would be 0° and supine on the floor would be 90°, using the following equations:

(3) $${\mathrm{Deg}}_{Saggital}=\tan^{-1}\frac{ACC_{z}}{ACC_{y}}\times \frac{180}{\pi }$$

(4) $${\mathrm{Deg}}_{Frontal}=\tan^{-1}\frac{ACC_{x}}{ACC_{y}}\times \frac{180}{\pi }$$

**Figure 2:**
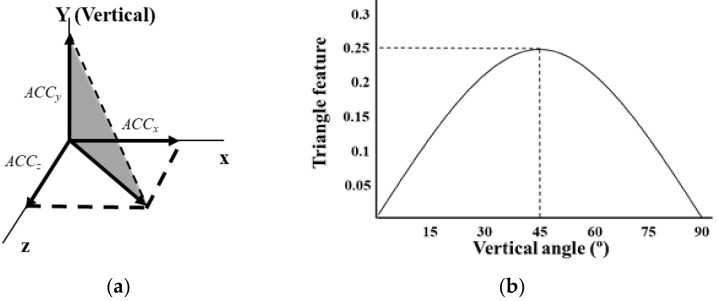
(**a**) Definition of triangle feature; (**b**) relationship between the vertical angle and the triangle feature.

The triangle feature was defined as the area of the triangle defined by the vector sum of the acceleration along the x- and z-axes and acceleration along the y-axis (Figure 2a). This area rapidly increased until the subject vertical angle reached 45°, and then decreased (Figure 2b). During standing, $ACC_{x}$, $ACC_{y}$ and, $ACC_{z}$ become 0 g, 1 g, and 0 g, respectively; thus, the triangle feature becomes 0. After the fall, $ACC_{x}$, $ACC_{y}$, and $ACC_{z}$ become 0 g, 0 g, and 1 g, respectively; thus, the triangle feature becomes 0. When the vertical angle is 45°, $ACC_{x}$, $ACC_{y}$, and $ACC_{z}$ are 0 g, $\frac{1}{\sqrt{2}}$ g, and $\frac{1}{\sqrt{2}}$ g, respectively; thus, the triangle feature becomes 0.25. The triangle feature according to the vertical angle is shown in Figure 2b.

### 2.5. Pre-Impact Fall Detection Algorithm

The pre-impact fall detection algorithm was applied to falls and ADLs performed by 30 subjects. All data were low-pass filtered at 8 Hz. Acceleration, angular velocity, vertical angle, and the triangle feature were used as threshold values to define falls in the algorithm. Determining optimal thresholds of these factors when defining falls is difficult. Too low a threshold may yield many false positives by detecting falls during ADLs, resulting in lower specificity. Too high a threshold may yield many false negatives by failing to detect falls, resulting in lower sensitivity. Therefore, sensitivity, specificity, and accuracy were calculated for all possible thresholds based on experimental data in order to optimize the thresholds.

Sensitivity, specificity, and accuracy are respectively defined as:

(5) $$Sensitivity\left(\%\right)=\frac{True\;positives}{True\;positives+False\;negatives}\times 100$$

(6) $$Specificity\left(\%\right)=\frac{True\;negatives}{True\;negatives+False\;positives}\times 100$$

(7) $$Accuracy\left(\%\right)=\frac{True\;positives+True\;negatives}{True\;positives+True\;negatives+False\;positives+False\;negatives\;}\times 100$$

where *True positives* denotes a fall occurs and the algorithm detects it, *False positives* means ADLs (no fall) and algorithm detects a fall, *True negatives* denotes ADLs (no fall) and algorithm does not detect a fall, and *False negatives* means a fall occurs and the algorithm does not detect it.

**Figure 3:**
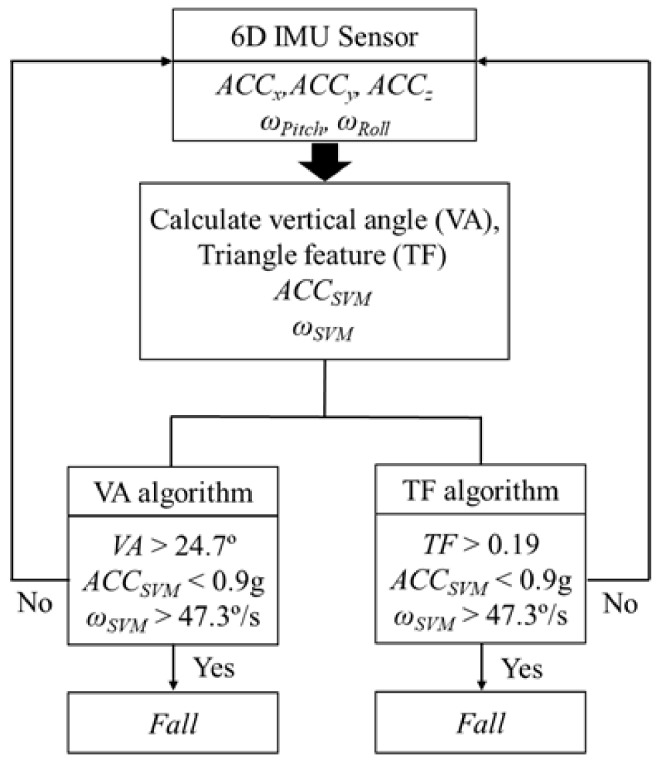
Pre-impact fall detection algorithm (Vertical angle (VA) and Triangle feature (TF) algorithms).

Lead time was defined as the time between fall detection and impact [14,15]. The impact was defined as the collision of the hip with the ground. Many thresholds with 100% accuracy were obtained from the optimization, and the one that maximized the lead time was selected for the detection algorithm. Results showed that a fall was detected based on the VA algorithm when the vector sum of the acceleration was less than 0.9 g, the angular velocity was greater than 47.3°/s, and the vertical angle was greater than 24.7° (Figure 3). Similarly, a fall was detected based on the TF algorithm when the vector sum of acceleration was less than 0.9 g, the angular velocity was greater than 47.3°/s, and the triangle feature was larger than 0.19 (Figure 3).

**Figure 4:**
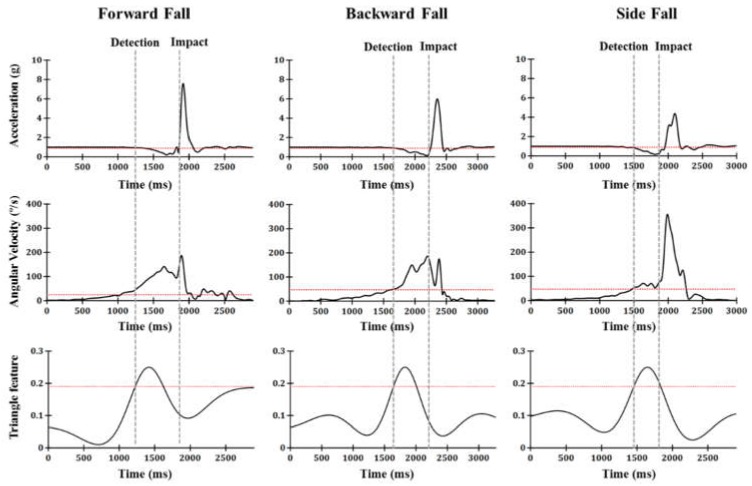
(**top**) Acceleration, (**middle**) angular velocity, and (**bottom**) triangle feature data during falls.

Inertial properties (acceleration, angular velocity, and triangle feature) during falls are shown in Figure 4. The angular velocity increased first and passed the threshold value of 30°/s. Next, the acceleration started to decrease from 1 g and passed the threshold value of 0.9 g. Finally, the triangle feature, which is the tilting characteristic of the body, exceeded the threshold value of 0.19, and then the fall was detected before the impact.

### 2.6. Algorithm Evaluation Using Public Dataset

Two algorithms were developed in this study based on the data only from younger subjects. These algorithms might not be suited for real-world use in elderly fall injury prevention because, on average, younger people move differently than older people, who have slower reaction times and less ability to rescue themselves from falling. The performance of the algorithm, using vertical angle (VA) and triangle feature (TF), was evaluated using the SisFall dataset. The SisFall dataset were selected based on the survey [29], as they better reflect reality than many previous fall experiments [25,26,27,28]. Table 2 and Table 3 show the falls and ADLs that the subjects engaged in to create the SisFall dataset.

## 3. Results

### 3.1. Sensitivity and Specificity

Both the VA and TF algorithms were implemented for 10 subjects. Neither algorithm failed to detect any of the four types of falls during any trial, which meant that both algorithms had 100% sensitivity. Neither algorithm improperly detected a fall during any trial of the six different types of ADLs, which meant that both algorithms had 100% specificity.

The two algorithms developed in this study were evaluated using the SisFall dataset. Both algorithms successfully detected all falls, representing 100% sensitivity. Table 4 shows the number of false positives during ADLs. The VA and TF algorithms showed specificities of 78.3% and 83.9%, respectively.

### 3.2. Lead Time

The lead times generated by each algorithm for each of the four different types of falls are listed in Table 5. The mean lead times of the TF and VA algorithms were 427 ± 45.9 ms and 401.9 ± 46.9 ms, respectively.

### 3.3. Comparison with Other Studies

Table 6 compares the performance of the VA and TF algorithms with those from previous studies. The TF algorithm showed 90.3% accuracy (100% sensitivity and 83.9% specificity), and the VA algorithm showed 86.9% accuracy (100% sensitivity and 78.3% specificity).

## 4. Discussion

In this study, pre-impact fall detection algorithms were developed using an IMU sensor positioned at subjects’ waists. The algorithms used acceleration, angular velocity, the subjects’ vertical angles, and triangle features to detect falls.

Many studies have used pre-impact fall detection algorithms to trigger wearable fall-injury minimization systems [30,31,32,33]. However, the accuracy of these systems has been low. Some studies have been able to achieve 100% specificity, but not 100% sensitivity [15,17]. These systems were particularly prone to false-positive errors or registering jumps or standing-to-sitting transitions as falls. If acceleration was used as the only threshold, jumping and sitting in a chair could be mistaken for falling. The algorithms developed in this study used subjects’ vertical angles and triangle features in addition to acceleration and angular velocity to avoid false-positive errors.

Thelen et al. [34] found that the average maximum lean angle from which subjects could recover their balance with a single forward step was 32.5° for younger men and 23.9° for older men. Based on these findings, the VA threshold of 24.7° used in this study was well within the limit for balance recovery for younger men and close to the limit for older men.

For ADLs that involved lying down in this study, only a small variation was observed in acceleration, so the algorithms did not improperly detect any falls. Fall detection systems can improperly detect falls when subjects are moving while lying down, such as while watching television or reading. A VA threshold of 60° was added at the beginning of the algorithm to avoid these mistakes. However, the TF algorithm did not improperly detect falls while subjects were lying down because it has a feature that TF decreased when tilt exceeded 45° and did not exceed the threshold when lying down. The TF algorithm was able to detect falls at least 30 ms earlier than the VA algorithm because the triangle feature passed its threshold more quickly than the vertical angle.

Although many fall detection algorithms have been developed, they have not been put to practical use because they were not developed using ADL and fall data from elderly subjects [14,15,16,17,18,19,20]. Publicly available datasets were generated from a few activities and none included fall data from elderly subjects [25,26,27,28]. However, the SisFall dataset [29] was recently made publicly available and was developed based on data from more participants, more ADLs, and more falls than any other publicly available dataset [25,26,27,28]. The SisFall dataset contains data from elderly people. In this study, the performance of two algorithms (VA algorithm and TF algorithm) was evaluated using the SisFall dataset [29]. 

The VA and TF algorithms developed in this study were evaluated using the SisFall dataset. All falls were detected by each algorithm, meaning that both had 100% sensitivity. However, the F10 and F13 falls were only detected after impact because the subjects’ knees hit the ground. The algorithms created in the previous study were intended for use in systems to protect hip joints, so these failures to detect falls until after impact did not count against the usefulness of the algorithms in this context. The VA algorithm had a specificity of 78.3% because it generated false positives for the D08, D09, D10, D12, D13, and D17 ADLs (Table 4). The TF algorithm had a specificity of 83.9% because it generated false positives for the D10, D13, and D17 ADLs (Table 4). These ADLs involved transitions to lower positions, such as sitting in a low chair quickly, lying down quickly, or getting in a car. New features need to be added to the algorithms to eliminate these false positives. However, these algorithms were more accurate than the one previously reported [30]. The TF algorithm had higher specificity than the VA algorithm; the TF algorithm performed better than VA algorithm for ADLs that involved lower postures because it decreased when the slope exceeded 45°.

The results revealed that the VA and TF algorithms had higher specificity for elderly subjects than for younger subjects. Elderly people generally move slower than younger people because they are relatively weaker and have relatively lower motor ability and sensory function than younger people [34,35]. It is more advantageous to be able to detect elderly peoples’ falls because of the large difference in speed between ADLs and falls.

Lead time was an important factor in evaluating the effectiveness of the VA and TF algorithms. Lead time was calculated as the duration between the time that the algorithm detected a fall and the time at which the subject’s hip impacted the ground, as detected using a force plate or video equipment [16]. However, accurate lead times could not be calculated from the SisFall dataset because the impact time was not provided.

## 5. Conclusions

In this study, pre-impact fall detection algorithms were developed using an inertial sensor unit. VA and TF algorithms detected falls with 100% accuracy with our dataset and resulted in lead times of 401 ± 46.9 ms and 427 ± 45.9 ms, respectively. The performance of the algorithms was evaluated using the SisFall dataset [29]. The developed algorithms were shown to be more accurate than existing algorithms. Improved fall detection would significantly improve the value of fall protection systems to protect the elderly from injuries sustained during falls.

## Figures and Tables

**Table 1:** Description of activities. ADL = activities of daily living.

	Activity	Description
**ADLs**	Sit-to-stand	Standing up slowly from the stool
	Walking	Walking straight along the line of the floor
	Stand-to-sit	Slowly sitting in a stool
	Sit-to-lie	Sitting at the end of the mattress, then laying down in a natural motion
	Jumping	Jumping to the maximum height in place
	Running	Running straight along the line of the floor
**Falls**	Forward fall	Fainting fall in the forward direction
	Backward fall	Fainting fall in the backward direction
	Side fall	Fainting fall in the lateral direction
	Twist fall	Rotating about the vertical axis during the backward fall

**Table 2:** Types of falls in SisFall dataset.

Code	Activity	Trials	Duration (s)
**F01**	Fall forward while walking caused by a slip	5	15
**F02**	Fall backward while walking caused by a slip	5	15
**F03**	Lateral fall while walking caused by a slip	5	15
**F04**	Fall forward while walking caused by a trip	5	15
**F05**	Fall forward while jogging caused by a trip	5	15
**F06**	Vertical fall while walking caused by fainting	5	15
**F07**	Fall while walking, with use of hands in a table to dampen fall, caused by fainting	5	15
**F08**	Fall forward when trying to get up	5	15
**F09**	Lateral fall when trying to get up	5	15
**F10**	Fall forward when trying to sit down	5	15
**F11**	Fall backward when trying to sit down	5	15
**F12**	Lateral fall when trying to sit down	5	15
**F13**	Fall forward while sitting, caused by fainting or falling asleep	5	15
**F14**	Fall backward while sitting, caused by fainting or falling asleep	5	15
**F15**	Lateral fall while sitting, caused by fainting or falling asleep	5	15

**Table 3:** Types of ADLs in SisFall dataset.

Code	Activity	Trials	Duration (s)
**D01**	Walking slowly	1	100
**D02**	Walking quickly	1	100
**D03**	Jogging slowly	1	100
**D04**	Jogging quickly	1	100
**D05**	Walking upstairs and downstairs slowly	5	25
**D06**	Walking upstairs and downstairs quickly	5	25
**D07**	Slowly sit in a half height chair, wait a moment, and up slowly	5	12
**D08**	Quickly sit in a half height chair, wait a moment, and up quickly	5	12
**D09**	Slowly sit in a low height chair, wait a moment, and up slowly	5	12
**D10**	Quickly sit in a low height chair, wait a moment, and up quickly	5	12
**D11**	Sitting a moment, trying to get up, and collapse into a chair	5	12
**D12**	Sitting a moment, lying slowly, wait a moment, and sit again	5	12
**D13**	Sitting a moment, lying quickly, wait a moment, and sit again	5	12
**D14**	Being on one’s back change to lateral position, wait a moment, and change to one’s back	5	12
**D15**	Standing, slowly bending at knees, and getting up	5	12
**D16**	Standing, slowly bending without bending knees, and getting up D17	5	12
**D17**	Standing, get into a car, remain seated and get out of the car	5	25
**D18**	Stumble while walking	5	12
**D19**	Gently jump without falling (trying to reach a high object)	5	12

**Table 4:** Number of false positives that occurred during ADLs.

Code	VA Algorithm		TF Algorithm	
	Young	Elderly	Young	Elderly
**D01−D05**	0/23	0/15	0/23	0/15
**D05, D07, D11, D14−D16**	0/115	0/75	0/115	0/75
**D06, D18, D19**	0/115	0/5	0/115	0/5
**D08**	32/115	13/75	0/115	0/75
**D09**	28/115	17/75	0/115	0/75
**D10**	115/115	75/75	115/115	75/75
**D12**	27/115	21/75	0/115	0/75
**D13**	115/115	5/5	115/115	5/5
**D17**	115/115	29/75	115/115	12/75

**Table 5:** Lead times based on VA and TF algorithms.

Type of Fall	VA Algorithm (ms)	TF Algorithm (ms)
**Forward Fall**	403 ± 32.7	423 ± 22.8
**Side Fall**	422 ± 42.3	422 ± 31.8
**Backward Fall**	423 ± 33.1	442 ± 47.4
**Twist Fall**	381 ± 19.0	397 ± 27.8
**Mean ± SD**	401 ± 46.9	427 ± 45.9

**Table 6:** Accuracy comparison with previous studies.

	Wu [17]	Tamura et al. [31]	Bourke et al. [15]	This Study	
				VA Algorithm	TF Algorithm
Accuracy (%)	80.5	81.8	87.2	86.9	90.3
Sensitivity (%)	100	93	100	100	100
Specificity (%)	67.6	74.4	78.7	78.3	83.9
Feature	Acceleration	Acceleration Angular velocity	Vertical velocity	Acceleration Angular velocity Vertical angle	Acceleration Angular velocity Triangle feature
